# IL-13 is a therapeutic target in radiation lung injury

**DOI:** 10.1038/srep39714

**Published:** 2016-12-22

**Authors:** Su I. Chung, Jason A. Horton, Thirumalai R. Ramalingam, Ayla O. White, Eun Joo Chung, Kathryn E. Hudak, Bradley T. Scroggins, Joseph R. Arron, Thomas A. Wynn, Deborah E. Citrin

**Affiliations:** 1 Radiation Oncology Branch, Center for Cancer Research, National Institutes of Health, Bethesda, Maryland, USA; 2 Musculoskeletal Science Research Center, Dept. of Orthopedic Surgery, Upstate Medical University, Syracuse, New York, USA; 3 Biomarker Discovery OMNI, Genentech, Inc. MS 231c, 1 DNA way, San Francisco, CA 94080 USA; 4 Laboratory of Parasitic Diseases, National Institute of Allergy and Infectious Diseases, 4 Memorial Drive, Room 211C, Bethesda, MD 20892-0425, USA

## Abstract

Pulmonary fibrosis is a potentially lethal late adverse event of thoracic irradiation. Prior research indicates that unrestrained TGF-β1 and/or type 2 cytokine-driven immune responses promote fibrosis following radiation injury, but the full spectrum of factors governing this pathology remains unclear. Interleukin 13 (IL-13) is a key factor in fibrotic disease associated with helminth infection, but it is unclear whether it plays a similar role in radiation-induced lung fibrosis. Using a mouse model, we tested the hypothesis that IL-13 drives the progression of radiation-induced pulmonary fibrosis. Irradiated lungs from wild-type c57BL/6NcR mice accumulated alternatively-activated macrophages, displayed elevated levels of IL-13, and extensive fibrosis, whereas IL-13 deficient mice were resistant to these changes. Furthermore, plasma from irradiated wild-type mice showed a transient increase in the IL-13 saturated fraction of the circulating decoy receptor IL-13Rα2. Finally, we determined that therapeutic neutralization of IL-13, during the period of IL-13Rα2 saturation was sufficient to protect mice from lung fibrosis. Taken together, our results demonstrate that IL-13 is a major regulator of radiation-induced lung injury and demonstrates that strategies focusing on IL-13 may be useful in screening for timely delivery of anti-IL-13 therapeutics.

Radiation-induced lung injury occurs in patients exposed to therapeutic radiation for hematopoietic transplant conditioning or for treatment of breast and thoracic malignancies. In developing a treatment plan, radiation oncologists must balance the likelihood of delivering a curative dose of radiation against the risk of normal tissue toxicity. Acute toxicity experienced during treatment can be addressed by altering the prescribed radiation dose and/or delivery schedule, but late-developing adverse events including fibrosis may arise weeks to years following completion of radiotherapy. It is difficult to determine, *a priori*, which patients will develop pulmonary fibrosis following irradiation, yet as many as 43% of patients will display radiographic evidence of lung injury^1^, and 15% of treated patients becoming symptomatic^2^. Presently, there are no clinically useful diagnostic markers or FDA approved mitigant or treatment strategies for radiation fibrosis.

Pulmonary fibrosis resulting from exposure to toxic agents, including radiation, is characterized by activation of stromal fibroblasts, infiltration of inflammatory cells, and unopposed progressive deposition of extracellular matrix. Collectively, this can lead to pulmonary failure due to impaired gas exchange and restrictive defects^3^. While there is a great deal of knowledge regarding the etiology of fibrotic diseases, the complex array of context-dependent, cytokine-driven pathways implicated in this pathology hinders progress toward effective interventions. The pro-fibrotic and pro-inflammatory mediators TGF-β, IL-1β, and IL-6 have been implicated as drivers of fibrosis after exposure to irradiation^4^,^5^, but the role of type 2 cytokines in promoting radiation lung injury remains unclear.

Type 2 cytokines such as IL-4, IL-10, and IL-13, are sufficient to induce non-polarized tissue resident macrophages to differentiate into pro-fibrotic, alternatively activated macrophages. Indeed, alternatively-activated macrophages, induced by type 2 associated cytokines have been reported to contribute to fibrotic responses to injury, such as those induced by parasitic infections^6^, fungal infections^7^, and, bleomycin^8^. Similarly, alternatively activated macrophages accumulate in radiation-induced fibrosis of other tissues such as skin^9^ suggesting that type 2 cytokines could contribute to alternative macrophage accumulation and pulmonary fibrosis following thoracic irradiation.

In the present study, we examined the role of type 2 cytokines, particularly IL-13, as mediators of fibrotic progression following radiation-induced lung injury. We evaluated the progression of radiation-induced pulmonary fibrosis in wild-type and IL-13-deficient mice, and characterized the inflammatory milieu in irradiated lung tissue, demonstrating that this cytokine is essential to the development of pulmonary fibrosis. Importantly, we discovered that the circulating level of IL-13 exceeded the inhibitory capacity of the soluble endogenous decoy receptor, IL-13Rα2 in irradiated mice, immediately prior to fibrotic progression. Using this information, we demonstrated that administration of an IL-13 neutralizing antibody can interrupt fibrotic progression following radiation injury. Thus, our findings provide evidence that IL-13 is a critical mediator of radiation lung injury, and may be a candidate biomarker and target for therapeutic intervention.

## Results

### Alternatively activated macrophages characterize the alveolar inflammatory infiltrate after irradiation

**Figure 1:**
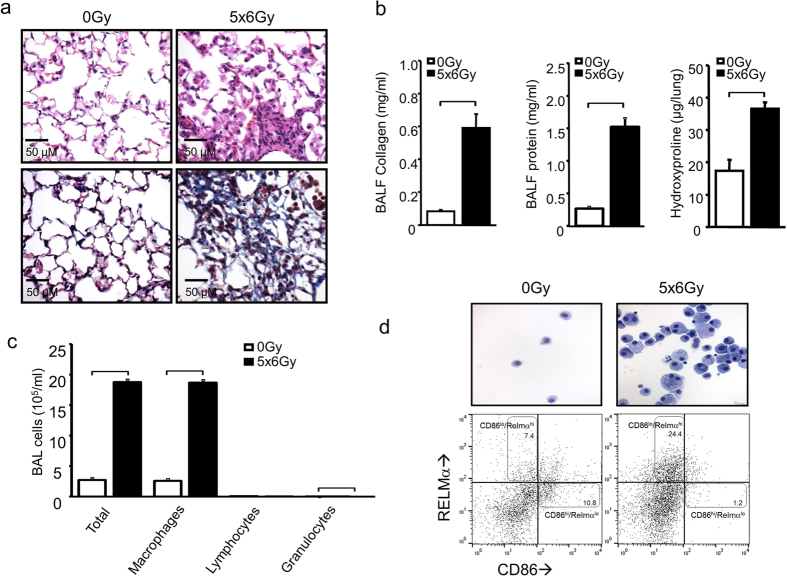
The effects of irradiation in murine lung. Eight to 10 week old female c57BL6/NcR mice were exposed to 5 daily fractions of 6 Gy (5 × 6 Gy) of thoracic irradiation. At 16 weeks after irradiation, lung tissue (n = 5 mice) and bronchoalveolar lavage (BAL) fluid (n = 5 mice) was collected from irradiated mice and controls (0 Gy). **(a)** Hematoxylin and Eosin (H&E) staining (top panels) and Masson Trichrome staining (bottom panels, collagen: light blue, epithelia: red, nuclei: dark blue) of lung demonstrates fibrotic foci. Bar: 50 μm. **(b)** Collagen and protein content of BAL fluid and hydroxyproline content of mouse lung were increased at 16 weeks after irradiation compared to unirradiated controls. **(c)** Cytospin of BAL fluid cellular content was stained with Diff-Quick and scored by phenotype. **(d)** Representative image of stained macrophages isolated from BAL fluid (upper panel). Flow cytometric analysis of CD86 and RELM-α expression with flow cytometry (lower panel). Columns: mean, error bars: +SD, brackets: p < 0.05; Student’s t-test.

Treatment of c57BL/6Ncr mice (wild type, WT) with 5 daily fractions of 6 Gy (5 × 6 Gy) to the thorax resulted in uniform lethality by 20 weeks after irradiation^10^. Evaluation of lung tissue at 16 weeks after irradiation revealed dense foci of sub-pleural fibrosis with collagen accumulation, alveolar thickening, and increased alveolar cellularity (Fig. 1a). Bronchoalveolar lavage (BAL) fluid from irradiated mice contained elevated levels of soluble collagen and protein, consistent with alveolar injury (Fig. 1b). Similarly, the hydroxyproline content of irradiated lung tissue was significantly increased relative to unirradiated control lungs (Fig. 1b). Assessment of the cellular composition of BAL fluid at 16 weeks after irradiation revealed that accumulation of macrophages is largely responsible for the increase in BAL cellularity (Fig. 1c,d). Furthermore, whereas classically activated (CD86^high^/RELMα^low^) macrophages predominated in BAL from unirradiated lungs, BAL from irradiated mice was largely populated by alternatively activated, CD86^low^/RELMα^high^ macrophages. This suggests that pulmonary radiation injury results in a shift in macrophage polarization toward the phenotype typically induced by the type 2 cytokines IL-4 and IL-13^11^.

**Figure 2:**
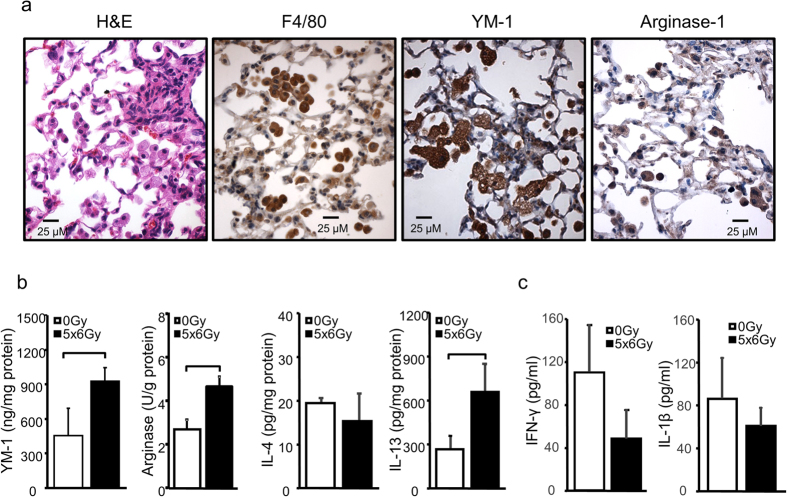
Type 2 inflammation in irradiated mouse lung. Eight-10 week old female c57BL6/NcR mice were exposed to 5 daily fractions of 6 Gy (5 × 6 Gy) of thoracic irradiation. **(a)** Sections of lung tissue collected at 16 weeks after irradiation or no irradiation (0 Gy) were immunostained for F4/80, YM-1, and Arginase-1. **(b)** Lung tissue (n = 5 mice) was homogenized and YM-1, IL-13, and IL-4 levels were measured with ELISA. Arginase-1 activity was also assessed. **(c)** Levels of interferon-γ and IL-1β in BAL fluid were measured with ELISA. Columns: mean, error bars: +SD, brackets: p < 0.05: Student’s t-test.

Immunohistochemistry demonstrated that the inflammatory infiltrates in irradiated lungs were largely composed of F4/80^+^ macrophages with high expression levels of the alternatively-activated macrophage markers Arginase-1 and YM-1 (Fig. 2a). Furthermore, lung homogenates had significantly increased levels of arginase activity and elevated concentrations of YM-1 (Fig. 2b). As these findings suggested an inflammatory environment dominated by type 2 cytokines, we also assayed levels of IL-4 and IL-13 in lung tissue homogenates. Interestingly, whereas we saw no difference in IL-4 levels, IL-13 concentration was significantly increased in irradiated lung tissue (Fig. 2b), a finding noted as early as two weeks after irradiation (Supplemental Fig. 1a). IL-13 expression was highest in fibrotic areas and tended to co-localize with intra-alveolar cells with the morphology of macrophages (Supplemental Fig. 1b). Levels of interferon-γ and IL-1β were not elevated in BAL fluid from irradiated mice compared to that in unirradiated control mice (Fig. 2c).

### Deficiency of IL-13 protects from radiation induced pulmonary fibrosis and death

**Figure 3:**
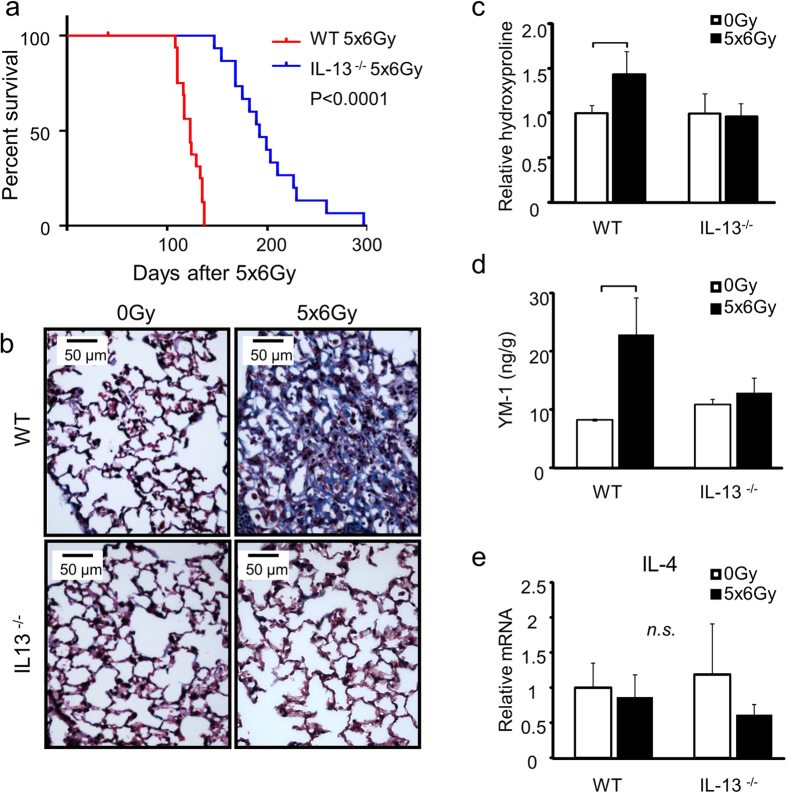
IL-13 deficiency protects against lethal radiation lung injury. Wild type mice (WT) or IL-13 deficient mice (IL-13^−/−^) were subjected to 5 × 6 Gy thoracic irradiation and followed for survival (n = 15) or tissue collection at 16 weeks after irradiation (n = 3–5). **(a)** IL-13^−/−^ mice have extended survival compared to WT mice following lethal thoracic irradiation. **(b)** Lung sections collected at 16 weeks after irradiation were subjected to Masson Trichrome staining. Dense fibrotic foci are evident in WT mice exposed to 5 × 6 Gy but are absent in the lungs of similarly treated IL-13^−/−^ mice. Hydroxyproline content **(c)**, YM-1 expression **(d)**, and IL-4 expression **(e)** were assessed in lung tissue from irradiated and unirradiated mice. Columns: mean, error bars: +SD, brackets: p < 0.05, n.s.: p ≥ 0.05; ANOVA with Tukey’s correction for non-survival comparisons, log rank for survival comparisons.

To determine whether IL-13 is a critical factor promoting radiation-induced pulmonary fibrosis we compared the survival duration of wild type and IL-13 deficient mice (IL-13^−/−^, c57BL/6NTac-[KO]IL13) exposed to thoracic irradiation (n = 15 per group, Fig. 3a). While wild type c57BL/6NcR mice had a median survival of 19.0 weeks post-irradiation, median survival time in IL-13-deficient mice was extended to 27.4 weeks (log rank test, p < 0.0001). Irradiated IL-13 deficient mice did not develop extensive pulmonary fibrosis (Fig. 3b), and displayed no significant change in hydroxyproline content (Fig. 3c) or YM-1 concentration (Fig. 3d) at 16 weeks after irradiation relative to unirradiated controls. Expression of IL-4 was not significantly altered in WT or IL-13 deficient mice after irradiation (Fig. 3e). These findings lend further support to the notion that IL-13 is a critical driver of radiation lung fibrosis.

**Figure 4:**
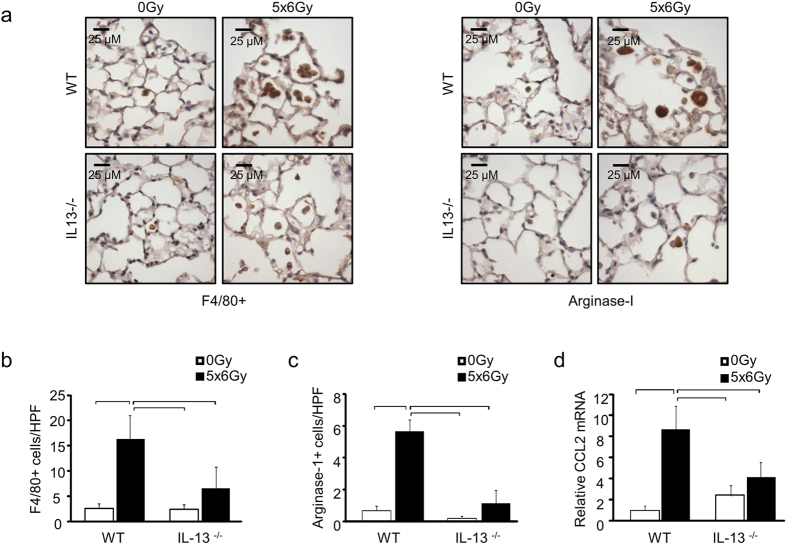
The effects of IL-13 deficiency on macrophage accumulation in irradiated lung. WT and IL-13^−/−^ mice (n = 3–5 mice) were subjected to 5 × 6 Gy thoracic irradiation or maintained unirradiated (0 Gy). F4/80 and Arginase-1 expression were assessed in lung tissue sections collected at 16 weeks after irradiation **(a**–**c)**. The number of stained cells per high power field (HPF, 40x) was scored in 6 fields per mouse. Lung tissue from IL-13^−/−^ mice demonstrated significantly reduced accumulation of cells expressing F4/80 and Arginase-1. **(d)** RT-PCR analysis of irradiated lung tissue demonstrated a significant reduction in radiation induced CCL2 expression in IL-13^−/−^ mice compared to WT lung tissue. Columns: mean, error bars: +SD, brackets: p < 0.05; ANOVA with Tukey’s correction.

We next sought to determine the mechanistic role that IL-13 plays in driving fibrosis, and whether the accumulation of alternatively activated macrophages was suppressed in IL-13 deficient mice after irradiation. Immunohistochemical assessment confirmed a reduction in infiltrating F4/80^+^ macrophages in the lungs of IL-13 deficient mice exposed to irradiation (Fig. 4a,b). Similarly, a reduction in Arginase-1 positive cells was observed in the lung tissue of IL-13 deficient mice exposed to irradiation compared to that of irradiated wild type control (Fig. 4a,c). The expression of CCL2, a chemoattractant factor for monocytes and macrophages, was induced in the lung tissue of irradiated wild type mice but was not significantly increased in the lung tissue of irradiated IL-13 deficient mice compared to unirradiated controls (Fig. 4d). Collectively, these findings lend support to the hypothesis that IL-13 drives type 2 inflammation after irradiation and confirm that deficiency of IL-13 is sufficient to prevent the accumulation of alternatively activated macrophages in the lung tissue of irradiated mice.

**Figure 5:**
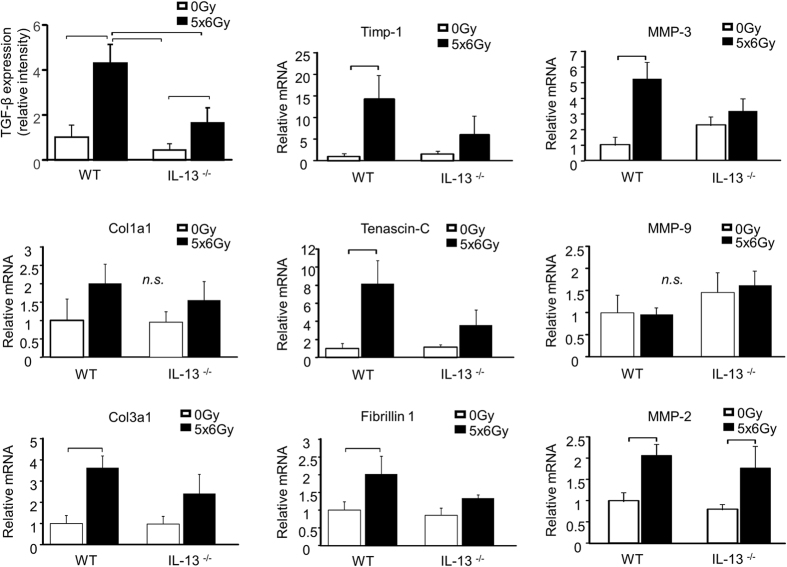
IL-13 deficiency prevents radiation induced expression of fibrosis associated genes. The expression of TGF-β, YM-1 protein, and the expression of fibrosis associated genes were assessed in irradiated and unirradiated (0 Gy) lung tissue at 16 weeks after exposure. IL-13^−/−^ mice were resistant to radiation induced expression of most fibrosis associated genes. Columns: mean, error bars: +SD, brackets: p < 0.05; Student’s t-test for comparisons within a genotype.

We next sought to determine if the function of IL-13 is additive to, or independent from, another pro-fibrotic cytokine implicated in radiation lung injury, TGF-β, by examining the expression of TGF-β and fibrosis-associated genes driven by TGF-β and IL-13. TGF-β was induced by irradiation in wild type lung tissue, but this response was blunted in IL-13 deficient lungs (Fig. 5). Smad2/smad3 phosphorylation was assessed by immunohistochemistry to provide confirmatory evidence of TGF-β signaling. Phosphorylated smad2/smad3 co-localized with TGF-β, and demonstrated a similar pattern of intensity as TGF-β (Supplemental Fig. 2). The expression of Col3a1, Timp-1, Tenascin-C, Fibrillin 1, MMP-3, and MMP-2 as assessed by RT-PCR was induced by irradiation in wild type lung tissue (Fig. 5). Consistent with a dominant role of IL-13 in radiation lung injury, the expression of these fibrosis-associated genes was not significantly increased after irradiation in IL-13 deficient mice, with the exception of MMP-2. The expression of MMP-9 and Col1a1 were not significantly increased by irradiation in our model at the 16 week time point. Collectively, these data confirm the importance of IL-13 in driving the expression of fibrosis-associated genes in irradiated lung tissue and suggest that TGF-β expression in irradiated lung tissue is reduced in the absence of IL-13 at this late time point.

### Thoracic irradiation suppresses circulating IL-13Rα2 resulting in saturation with IL-13

**Figure 6:**
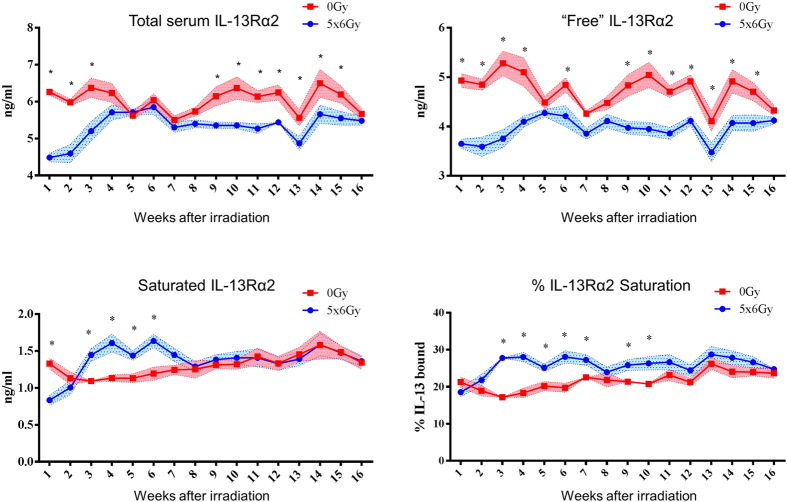
The effects of irradiation on IL-13Rα2 saturation. c57BL6/NcR mice were exposed to 5 × 6 Gy of thoracic irradiation. Sera from irradiated and unirradiated (0 Gy) mice (n = 5 mice per condition) were collected weekly for 16 weeks beginning at 1 week after irradiation. Total IL-13Rα2 levels were derived by adding the endogenously IL-13 bound IL-13Rα2 levels (‘saturated’) and ‘free’ IL-13Rα2 detected by *ex vivo* IL-13 saturation. Percent saturation is computed as [saturated IL-13Rα2/total IL-13Rα2] x 100. *p < 0.05. Statistical significance determined by t-tests, without correcting for multiple comparisons using an alpha of 5%.

As the high affinity soluble IL-13Rα2 decoy receptor prevents IL-13 from engaging the Stat6-activating IL-4Rα/IL-13Rα1 receptor complex^6^,^12^,^13^, we sought to assess the circulating concentration of IL-13Rα2 and the level of IL-13Rα2 saturation, as the amount of unbound IL-13Rα2 has a major impact on IL-13 effector function. Wild type mice (n = 5 per condition) underwent weekly blood collection for 16 weeks after thoracic irradiation to determine temporal changes in serum concentrations of sIL-13Rα2 and its saturation with IL-13. Total serum sIL-13Rα2 was significantly reduced as early as the first week after irradiation, and after a brief recovery at five through eight weeks, again became reduced compared to unirradiated controls (Fig. 6). The percent saturation of sIL-13Rα2 was increased significantly in irradiated mice between three and ten weeks after irradiation. We hypothesized that saturation of sIL-13Rα2 resulted in increased bioavailability of IL-13 at levels sufficient to initiate or promote progression of fibrosis.

### IL-13 neutralization during IL-13Rα2 saturation is sufficient to reduce radiation induced pulmonary fibrosis

**Figure 7:**
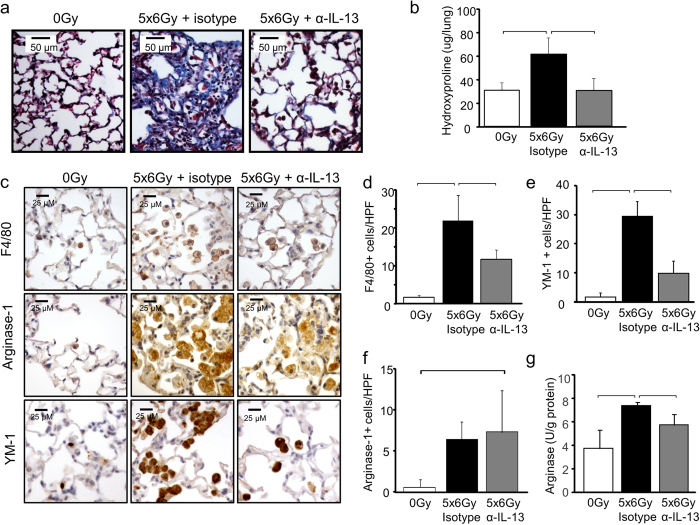
The effects of IL-13 neutralization on radiation induced lung injury. c57BL6/NcR mice were exposed to 5 × 6 Gy thoracic irradiation and treated with anti-IL-13 IgG or isotype control (0.5 mg per mouse) via IP injection. Additional mice were maintained without irradiation (0 Gy). Mice were treated weekly for eight weeks starting at 3 weeks post-irradiation, and lung tissue (n = 3 mice) was collected 16 weeks following irradiation. Collagen accumulation was assessed by Masson Trichrome staining **(a)** and hydroxyproline assay **(b)**. Anti-IL-13 treatment reduced radiation lung fibrosis. **(c**–**f)** F4/80, Arginase-1, and YM-1 expressing cells in lung sections (n = 3 mice) were detected with immunohistochemistry. The number of stained cells per high power field (HPF, 40x) was scored in 6 fields per mouse. **(g)** Arginase activity was assessed in lung tissue collected at 16 weeks after irradiation. Columns: mean, error bars: +SD, brackets: p < 0.05; ANOVA with Tukey’s correction.

Our previous data demonstrated that IL-13 is a major contributor to the progression of radiation-induced fibrosis, suggesting potential efficacy of strategies targeting IL-13. To confirm that systemic delivery of an IL-13 depleting agent was capable of preventing radiation fibrosis, and to validate that IL-13Rα2 saturation could guide the timing of therapy, irradiated wild type mice were treated with murine IgG targeting IL-13 (0.5 mg per animal) or an isotype control (Genentech, San Francisco, CA) with weekly dosing beginning at week 3 after irradiation and continuing through week 8. Treatment with anti-IL-13 IgG reduced collagen accumulation, histologic evidence of fibrosis, and macrophage accumulation (Fig. 7a,b,c). A marked reduction in the radiation-induced accumulation of F4/80^+^ cells in irradiated lung tissue was observed after anti-IL-13 IgG treatment (Fig. 7d). The accumulation of cells expressing YM-1 (Fig. 7e) or Arginase-1(Fig. 7f) was increased in irradiated lung, regardless of treatment.

**Figure 8:**
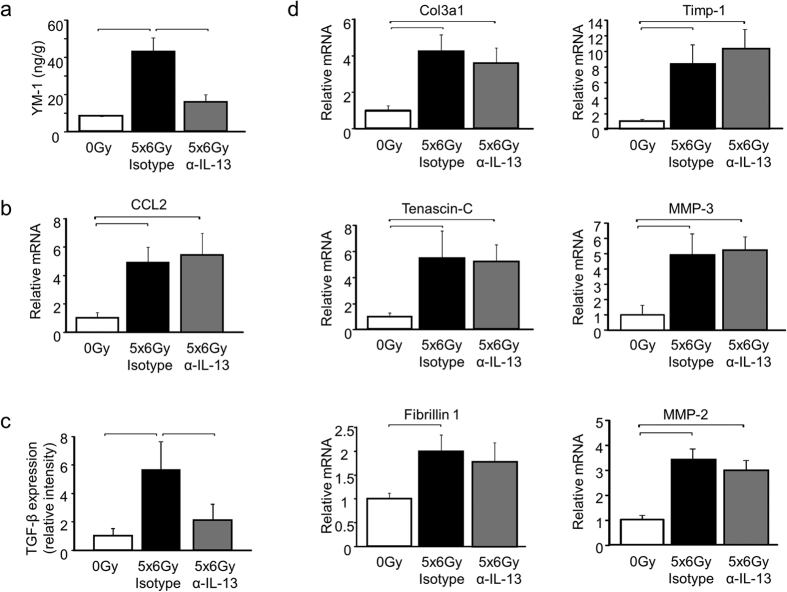
The effects of IL-13 neutralization on the expression of fibrosis associated genes. The expression of YM-1 protein (**a**), CCL2 mRNA (**b),** TGF-β (**c**) and the expression of fibrosis associated genes (**d**) was assessed in mice treated with either anti-IL-13 IgG or isotype control (n = 3 mice) at 16 weeks after 5 × 6 Gy of thoracic irradiation. YM-1 protein expression was suppressed in mice exposed to anti-IL-13 IgG compared to mice treated with the isotype control. The expression of fibrosis associated genes was similar in mice treated with anti-IL-13 IgG and the isotype control. Columns: mean, error bars: +SD, brackets: p < 0.05; ANOVA with Tukey’s correction.

Lung tissue collected at 16 weeks after irradiation (5 weeks after discontinuation of anti-IL-13 IgG treatment, n = 3 mice per treatment) exhibited reduced YM-1 concentrations and reduced Arginase activity in anti-IL-13 IgG treated mice compared to isotype control treated mice. The number of YM-1 labeled cells was markedly reduced in anti-IL-13 IgG treated mice compared to isotype treated controls. Although the total number of arignase-1 labeled cells was not statistically different between the two treatments, there was evidence of reduced intensity of arginase -1 staining in many regions of the lung. Collectively, these data suggest that the antibody inhibited recruitment and polarization of alternatively activated YM-1 positive macrophages (Fig. 7c,e and Fig. 8a). In contrast, the radiation-induced expression of CCL2 was not significantly reduced by anti-IL-13 neutralizing antibodies 5 weeks after the termination of treatment (Fig. 8b). The observed pattern of CCL2 expression is consistent with induction after discontinuation of anti-IL-13 IgG treatment.

The expression of TGF-β was increased in irradiated mouse lungs compared to unirradiated controls regardless of antibody treatment type; however, the level of TGF-β expression and smad2/smad3 phosphorylation were significantly lower in the lungs of mice treated with the IL-13 neutralizing antibody compared to those treated with the isotype antibody (Fig. 8c, Supplemental Fig. 3). In contrast, the fibrosis associated genes Col3a1, Tenascin-C, Fibrillin 1, TIMP-1, MMP-3, and MMP-2 were elevated in irradiated mouse lung, regardless of antibody treatment type (Fig. 8d). Collectively, these data suggest that time-targeted delivery of IL-13 neutralizing antibodies restricted to the period of increased IL-13Rα2 saturation provides a substantial reduction in fibrosis but incomplete protection against type 2 related inflammation in irradiated lung.

## Discussion

The pathologic mechanism of fibrosis can vary by the initiating insult and the organ in which the insult occurs. Radiation lung injury is associated with chronic inflammation, perpetuated by a cascade of cytokines that initiate an irreversible progression of fibrosis^4^,^5^,^14^. In our study, we sought to characterize the inflammatory milieu of the irradiated lung that leads to fibrotic progression. Infiltration of predominantly alternatively-activated macrophages, characterized by RELMα and YM-1 expression and Arginase-1, predominated in the alveolar space following irradiation. This finding strongly suggested the presence of type 2 polarized inflammation, previously associated with parasite-induced fibrosis^15^,^16^. However, the involvement of this immunologic program in the context of radiation lung injury and sterile inflammation remains largely unexplored. Our present study identifies IL-13 as a critical mediator of radiation-induced lung injury, with potential diagnostic and therapeutic importance.

IL-13 is a cytokine that has been implicated in type 2 driven inflammatory processes in several pulmonary diseases including parasitic, viral, and fungal infections^15^,^17^,^18^,^19^, as well as allergic diseases^20^,^21^, asthma^22^, bronchiolitis obliterans^23^,^24^, usual interstitial pneumonia^25^, and idiopathic pulmonary fibrosis. In irradiated mice, we observed increased IL-13 expression in lung tissue and BAL fluid at sixteen weeks after irradiation, consistent with the other markers of type 2 inflammation. Our observation that IL-13 deficient mice are exceptionally resistant to radiation-induced pulmonary fibrosis provides a strong rationale for therapeutic strategies targeting IL-13 to mitigate radiation lung injury. Furthermore, our study revealed that evaluation of circulating IL-13Rα2 concentration and saturation may be useful to understand the kinetics of IL-13 elaboration in injury models and to appropriately time anti-IL-13 interventions. Indeed, several investigators have previously studied the anti-fibrotic efficacy of targeting IL-13-induced signaling, either through targeted delivery of immunotoxins^8^, disruption of ligand-receptor interactions^26^,^27^,^28^, or by direct IL-13 neutralization, a strategy that is currently yielding promising results in humanized models^29^, and has advanced to clinical trials in human subjects with severe asthmatic airway disease and other disease driven by IL-13^30^,^31^,^32^,^33^. In our experiments, we used an IL-13 neutralizing IgG during the period of IL-13Rα2 nadir and saturation significantly disrupted the progression of radiation-induced pulmonary fibrosis. Prior studies^7^,^8^ have shown that timely inhibition of IL-13-mediated signaling is an important factor in effective mitigation of fibrotic disease induced by intratracheal bleomycin instillation or by pulmonary infection with *P aeuroginosa* or *A. fumigatus*. Albeit on a different temporal scale, likely reflecting the latency of fibrotic disease induced by ionizing radiation, our current data are consistent with the notion that anti-IL-13 therapeutics may be useful mitigants of progressive fibrotic lung disease. Importantly, our data suggests that diagnostic assessment of circulating bioactive IL-13 may prompt timely delivery of anti-IL-13 therapeutics during the period at which they may be most effective.

Conflicting data exists regarding the importance of IL-13 in TGF-β driven fibrosis. Previous work has demonstrated that TGF-β production and MMP-9 activity drive IL-13 mediated pulmonary fibrosis^34^,^35^. Indeed, inhibition or deficiency of IL-13 is sufficient to prevent TGF-β induction and fibrosis after bleomycin exposure^35^. Conversely, data from parasitic fibrosis models in liver have demonstrated complete abrogation of fibrosis in IL-13 deficient mice despite undiminished TGF-β production^36^. These data demonstrate the complexity of the dependence of IL-13 and TGF-β in fibrotic progression, a relationship that may be highly dependent of the pathologic insult and biologic context.

The interplay and independence of TGF-β and IL-13 in radiation lung injury are highlighted in our findings. TGF-β is considered a major driver of radiation-induced lung fibrosis^37^. We observed that IL-13 deficient mice were highly resistant to radiation-induced fibrosis compared to wild type mice. The observation that lung from IL-13 deficient mice exhibited a suppression in radiation-induced expression of TGF-β and TGF-β-responsive fibrosis-associated genes further demonstrates the important role IL-13 in modulating TGF-β responsive pathways necessary for fibrotic progression.

In contrast, mice treated with an anti-IL-13 IgG did not exhibit complete suppression of radiation-induced TGF-β expression and smad2/smad3 phosphorylation at 16 weeks after radiation exposure when compared to irradiated controls. One possible explanation is incomplete disruption of signaling in lung tissue though inadequate delivery. The decrease in fibrosis in irradiated lung observed after treatment with anti-IL-13 IgG supports an alternative explanation in which treatment discontinuation resulted in rebound of type 2 inflammation or TGF-β expression and down-stream fibrosis associated genes. Further study of parallel mechanisms in the setting of IL-13 neutralization, such as parenchymal senescence and chronic oxidative stress^10^, may reveal additional opportunities for intervention that complement neutralization of IL-13.

Circulating sIL-13Rα2, a decoy receptor, scavenges circulating IL-13 and reduces its engagement with the Stat6-activating IL-4Rα/IL-13Rα1 complex^38^. Our observation that circulating sIL-13Rα2 became saturated with IL-13 after irradiation suggested that systemic delivery of an IL-13 neutralizing agent may have therapeutic efficacy. In this study, the saturation of sIL-13Rα2 was used to guide therapy, based on the assumption that sIL-13Rα2 functions in a scavenging role. Although this IL-13Rα2 dynamic cannot be observed in humans owing to the absence of IL-13Rα2 splice variant that encodes the soluble isoform^39^, it is possible that serial monitoring of circulating or lavaged IL-13 in patients at substantial risk for lung injury will allow selection of appropriate subjects and optimal timing of IL-13 targeted therapy after thoracic irradiation. Alternatively, circulating levels of IL-13 responsive downstream biomarkers such as periostin, might serve as surrogates to stratify therapy^33^.

While effective at reducing fibrosis, treatment with an IL-13 neutralizing antibody did not completely suppress radiation-induced TGF-β expression or the expression of TGF-β regulated genes in irradiated lungs, a finding that does not confirm this regulatory hierarchy of IL-13 driving TGF-β responses. It is possible that either the antibody could not interfere with local signaling completely, or that treatment discontinuation resulted in rebound of TGF-β expression and down-stream fibrosis associated genes. Indeed, a rebound effect was noted following withdrawal of anti-TGF-β antibody treatment^40^. This rebound in fibrosis associated signaling may parallel the responses we observed following withdrawal of the anti-IL-13 IgG, and may suggest that sustained IL-13 or TGF-β neutralization therapy may be necessary to durably abrogate fibrotic progression^40^. Combinatorial strategies targeting these rebound pathways or other pathways implicated in fibrotic progression, such as parenchymal senescence and chronic oxidative stress^10^, may reveal additional opportunities for intervention that complement neutralization of IL-13.

The role of membrane bound and soluble forms of IL-13Rα2 in chronic radiation injury is unexplored. We observed a reduction in circulating sIL-13Rα2 after thoracic irradiation, particularly in the immediate post-irradiation window. A prior study has demonstrated increased IL-13Rα2 expression in irradiated intestine, which was hypothesized to play a role in intestinal epithelial repopulation^41^, however the elaboration of sIL-13Rα2 was not examined. Secretion of sIL-13Rα2 has been attributed to alternative splicing^42^, although protease MMP-8 mediated shedding of the membrane isoform has also been observed^43^. The cause and effects of the observed reduction in sIL-13Rα2 after irradiation of lung remains an area of active study.

Radiation injury in any organ is considered exceedingly difficult to treat due to the chronic progressive nature of the process. Indeed, there are no FDA-approved pharmacologic agents for treatment of radiation-induced fibrosis. Some clinical benefit has been observed with long term therapy with pentoxifylline and Vitamin E in patients with cutaneous radiation fibrosis^44^,^45^. Similarly, a small reduction in lung injury after irradiation has been observed in lung cancer patients treated with pentoxifylline in a randomized trial, however no benefit in survival was evident^46^. As a result of this marginal benefit, pentoxifylline treatment is not commonly used in the preventative setting for radiation fibrosis and is rarely used in the treatment of established radiation lung injury. The results of our study are particularly compelling given the numerous late-stage clinical trials targeting type 2 cytokines^32^,^33^,^47^,^48^,^49^, and the possibility for rapid clinical translation in the setting of radiation injury.

## Materials and Methods

### Mice and irradiation

All animal protocols and procedures were approved by the institutional Animal Care and Use Committee (National Cancer Institute, Bethesda, MD) and deemed in accordance with the guidelines of the Institute of Laboratory Animal Resources, National Research Council. c57BL/6NcR mice and IL-13 deficient mice (IL-13^−/−^, c57BL/6NTac-[KO]IL13) were obtained from Frederick National Laboratories (Frederick, MD) and NIAID/Taconic Biorepository, respectively. Eight to 10 week old female mice were used for all studies. Irradiation was performed with mice restrained in a custom Lucite jig with lead shielding that allowed for selective irradiation of the thorax. Five daily fractions of 6 Gy were delivered to the thorax with an X-RAD 320 (Precision X-Ray, North Branford, CT) using 2.0 mmAl filtration (320 kv peak) at a dose of 1.9 Gy/min.

### Antibody treatment

c57BL/6Ncr mice (n = 6) received a purified murine anti-IL-13 IgG antibody (Genentech, San Francisco, CA) or isotype control via intraperitoneal injection (0.5 mg per mouse weekly) for eight weeks beginning three weeks after irradiation.

### Tissue collection

Cohorts of mice were treated for survival analysis (n = 15) and tissue collection (n = 3–5 per condition). The right lung was collected and stored at −80 °C. The left lung was inflated with 10% neutral buffered formalin, paraffin embedded, and sectioned for histologic analysis. Weekly blood collection was performed (n = 5 mice per group) via mandibular bleeding. Serum was isolated and stored at −80 °C.

### BAL Collection and Analysis

Lungs from mice (n = 5 per condition) were pulsed with 1 ml ice cold 5 mM EDTA instilled via intratracheal catheter. The lavage was repeated once and pooled washings were centrifuged at 300 ×* g* for 10 minutes. The cell pellet was resuspended in 1 ml of ice cold PBS, and aliquoted for cell counting, cytospin, and flow cytometry. Cell counting was performed with a Countess device (Invitrogen). Differential cell counts were performed from cells prepared by cytospin (StatSpin Cytofuge2, Beckman-Coulter), stained with Diff-Quik (ThermoFisher, Waltham, MA), coverslipped, and mounted with Permount. At least 500 nucleated cells were counted per mouse and classified as granulocyte, lymphocyte, or monocyte/macrophage. For flow cytometry, cells were centrifuged, resuspended in 2% BSA, and labeled with APC conjugated CD86 (558703, BD Biosciences) and RELMα (ab39628, Abcam) antibodies; RELMα antibody was detected with Alexa Fluor 594 conjugated secondary antibody. Following staining the cells were washed, fixed with 1% paraformaldehyde, sorted on a FACSCalibur flow cytometer (BD Biosciences, Franklin Lakes, NJ) and analyzed with FlowJo software (Ashland, OR, USA).

Protein content of BAL supernatants (n = 5 per condition) was measured with a BCA assay (ThermoFisher, Waltham, MA). Soluble collagen content of the BAL fluid was assessed using a colorimetric dye-binding/elution assay. Briefly, 800 uL of Sirius Red/Picric Acid solution was added to 200 uL of BAL sample or standards, and mixed at room temperature for 1 hour. The collagen-dye complex was precipitated by centrifugation at 3,000 × g for 10 minutes. The pellets were washed in 0.5 M acetic acid and air-dried. The dye was eluted from the pellet in 1.5 mL of 0.5 M NaOH and the absorbance of each sample, blank, or standard was measured at 560 nm.

### Histopathology and histochemistry

Sections of embedded lung were depaffinized in xylene, rehydrated, and stained with hematoxylin and eosin or Masson trichrome stain (Sigma-Aldrich, St Louis, MO). Mounted slides were examined on a Leica DMRXA microscope (Wetzlar, Germany). Digital micrographs were captured at 10x magnification using a QImaging Micropublisher Camera (Surrey, BC, Canada).

For immunohistochemical staining, deparaffinized sections were subjected to antigen retrieval in citrate buffer pH 6.0 (Vector Labs, Burlingame, CA) in a pressure cooker. Sections were blocked with 2.5% normal horse serum for 1 hour and incubated with primary antibodies at 4 °C overnight. Endogenous peroxidase was quenched with 0.3% H_2_O_2_ for 10 minutes. Primary immunoreactivity was detected with a polymeric peroxidase-conjugated secondary antibody (ImmPress, Vector Labs) and visualized by 3,3′-diaminobenzidine histochemistry (ImmPACT, Vector). Sections were counterstained with Gill’s Hematoxylin (#3, Sigma-Aldrich), dehydrated, and mounted with Permount. F4/80 (ab6640) and Arginase-1 (ab11884) antibodies were purchased from Abcam (Cambridge, MA). YM-1 antibody was a gift from S. Kimura (NIH, Bethesda, MD).

For immunofluorescence staining, biotinylated anti-TGF-β and avidin-fluorescein were purchased from R&D Systems. Anti-phospho-Smad2/Smad3 (ab63399) was purchased from Abcam, and visualized with anti-Rabbit IgG conjugated with phycoerythrin. Labeled sections were counterstained with 4’,6-diamidino-2-phenylindole (DAPI) and mounted with Prolong Gold Anti-Fade Reagent (Life Technologies, Carlsbad, CA).

Images were captured as above at 10X–40X magnification. For quantitative assessments, the number of stained cells per high power field (HPF, 40x) was scored in 6 fields per mouse, 3 mice per condition. For quantitation of immunofluorescence, ten images (40x) were captured from 5 lungs for each condition and analyzed with ImageJ. Fluorescent intensity was determined for each channel in each image. The means for intensity for each condition was calculated, and the intensity for each condition was normalized to that of the unirradiated control.

### RNA extraction, cDNA synthesis, real time PCR

Total RNA from lung tissue was extracted with Trizol reagent (Life Technologies) and purified with the RNeasy plus system (Qiagen, Valencia, CA). 150–500 ng of RNA was reverse-transcribed using QuantiTect reverse transcription kit (Qiagen). Quantitative PCR (qPCR) was performed on an ABI 7500 system (Applied Biosystems) using Taqman gene expression assays (Life Technologies, Grand Island, NY).0.5–2 ul cDNA were amplified as follows: 50 °C for 2 min, 40 cycles of 95 °C for 15 s, and 60 °C for 1 min. The fold change in mRNA expression for targeted gene was expressed relative to that observed in lung tissue from WT unirradiated mice after normalization to actin.

### Enzyme-linked immunosorbent assay and Arginase Assay

Lung homogenates were prepared with ice-cold T-Per buffer (Thermo Scientific, Waltham, MA) containing 1x Halt Protease and Phosphatase inhibitor cocktail (Thermo Scientific). Equal protein concentrations were used for ELISA sandwich assays (R&D Systems, Minneapolis, MN) performed according to manufacturer’s recommended protocol. Arginase activity was assessed using the QuantiChrom Arginase assay kit (BioAssay Systems) as per manufacturer’s protocol. Equal volumes of tissue homogenate were used and arginase activity (units per gram protein) was normalized to total protein concentrations.

### Free and saturated IL-13Ra2 Assay

Kinetics of IL-13Rα2 levels in mouse sera were detected by ELISA, adapting a previously published method^50^. Briefly, microtiter plates were coated overnight with affinity-purified goat polyclonal anti-mouse IL-13Ra2 antibody (R&D systems). After blocking with 5% non-fat dry milk (Carnation), mouse serum samples (n = 5 per condition and time point) were added at 1:26 dilution. After washing with 0.05% PBS/Tween buffer, captured IL-13Rα2/IL-13 complexes (endogenously saturated) were secondarily bound with biotinylated monoclonal anti-IL-13 antibody (gift from Centocor), followed by detection with HRP-streptavidin conjugate and chromogenic substrate development using SureBlue Reserve (KPL). To detect free IL-13Rα2 captured from sera, replicate samples were saturated with 40 ng/ml of exogenous recombinant mouse IL-13 (gift from Pfizer) during sample incubation.

### Hydroxyproline Assay

Hydroxyproline content was measured using a colorimetric assay after hydrolysis of lung tissue in 6 N HCl at 110 °C for 18 h and subsequent neutralization in 6 N NaOH. Briefly, 20 μl hydrolysate was evaporated in 96 well plates after which 20 μl deionized water and 40 μl isopropanol were added. 20 μl of 1.4% chloramine T in citrate acetate buffer was then added and incubated for 30 minutes at room temperature, followed by the addition of 125 ul of 18.6% Ehrlich’s solution in isopropanol for 30 minutes at 65 °C. Optical density was read at 562 nm and corrected for background. A standard curve was used to calculate hydroxyproline concentration. Hydroxyproline was calculated based upon total lung weight and expressed as micrograms in the lung.

### Statistical Analysis

All data is represented as mean ± SD unless otherwise noted. Statistical analysis was performed using either Student’s t-test for comparisons between two conditions or ANOVA with Tukey’s correction for multiple comparisons. A p value of ≤0.05 was considered significant for comparisons. All studies in tissues were conducted in triplicate unless otherwise noted.

## Additional Information

**How to cite this article**: Chung, S. I. *et al*. IL-13 is a therapeutic target in radiation lung injury. *Sci. Rep.* **6**, 39714; doi: 10.1038/srep39714 (2016).

**Publisher's note:** Springer Nature remains neutral with regard to jurisdictional claims in published maps and institutional affiliations.

## Supplementary Material

Supplemental Figures
