# Supplementary material for: IL-13 is a therapeutic target in radiation lung injury
Source: Sci Rep. 2016 Dec 22;6:39714. doi: 10.1038/srep39714 (PMC5177927; doi:10.1038/srep39714)
Supplement: Supplemental Figures [file srep39714-s1.pdf]

IL-13 is a therapeutic target in radiation lung injury

Supplemental Data

Authors: Su I. Chung[1*†], Jason A. Horton[2*†], Thirumalai R. Ramalingam[3], Ayla O. White[1], Eun Joo Chung[1], Kathryn E. Hudak[1], Bradley T. Scroggins[1], Joseph R. Arron[3], Thomas A. Wynn[4], and Deborah E. Citrin[1*]

Affiliations:

[1] Radiation Oncology Branch, Center for Cancer Research, National Institutes of Health, Bethesda, Maryland.

[2] Musculoskeletal Science Research Center, Dept. of Orthopedic Surgery, Upstate Medical University, Syracuse, New York.

[3] Biomarker Discovery OMNI, Genentech, Inc. MS 231c, 1 DNA way, San Francisco, CA 94080. USA.

[4] Laboratory of Parasitic Diseases, National Institute of Allergy and Infectious Diseases, 4 Memorial Drive, Room 211C, Bethesda, MD 20892-0425, USA.

[†]Equally contributing

*****Corresponding Author:

Deborah Citrin, M.D.

Radiation Oncology Branch

National Cancer Institute

Building 10 CRC, B2-3500

Bethesda, MD

Telephone: 301-496-5457

Fax: 301-480-5439

E-mail: citrind@mail.nih.gov

Table of contents:

Supplemental Figure 1. IL-13 expression in irradiated lungs. **Page 4**

Supplemental Figure 2. Expression of TGF-β and phosphorylated smad2/smad3 in irradiated lungs. **Page 5**

Supplemental Figure 3. Effects of IL-13 neutralization on the expression of TGF-β and phosphorylated smad2/smad3 in irradiated lungs. **Page 6**

Supplemental Figure 4. Histologic findings in long-term survivors of thoracic irradiation. **Page 7**

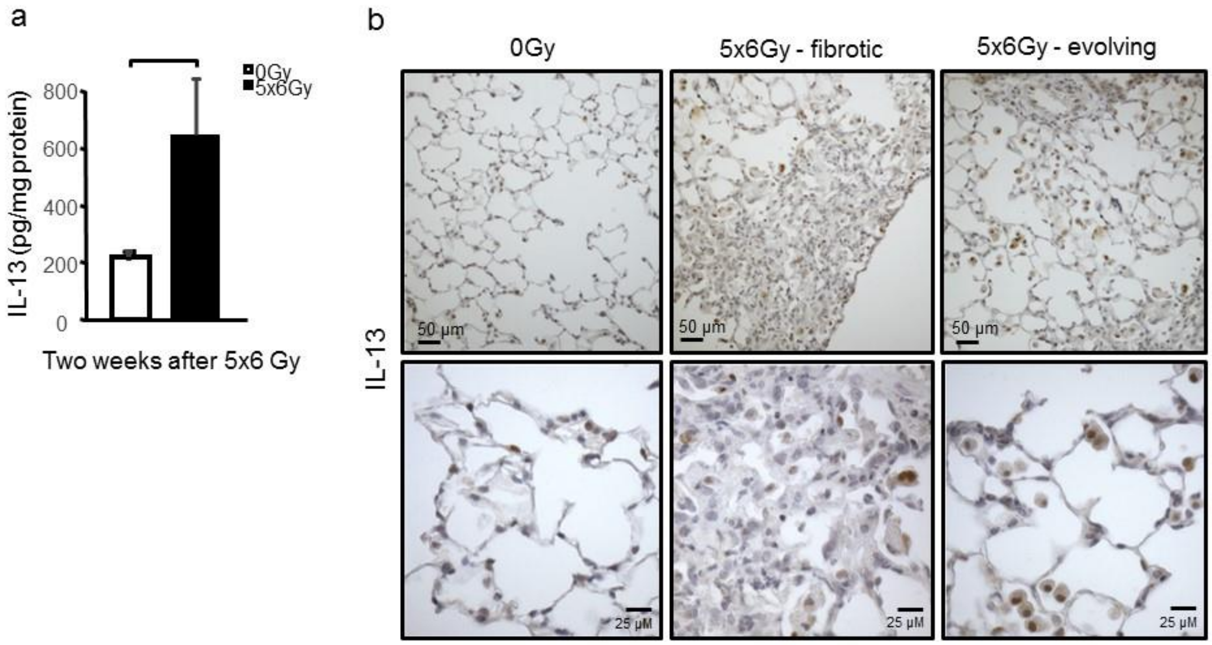

Supplemental Figure 1. IL-13 expression in irradiated lungs.

Eight to 10 week old female c57BL6/NcR mice were exposed to 5 daily fractions of 6 Gy (5x6Gy) of thoracic irradiation. **(a)** IL-13 protein levels were evaluated with ELISA in lung lysates collected at two weeks after irradiation. IL-13 levels were markedly increased in irradiated lung compared to unirradiated lung. **(b)** At 16 weeks after irradiation, lung tissue (n=5 mice per condition) was collected from irradiated mice and controls (0Gy). IL-13 expression was assessed with immunohistochemistry in formalin fixed, paraffin embedded tissue sections with DAB as the chromogen (brown) and hematoxylin counterstain (blue).

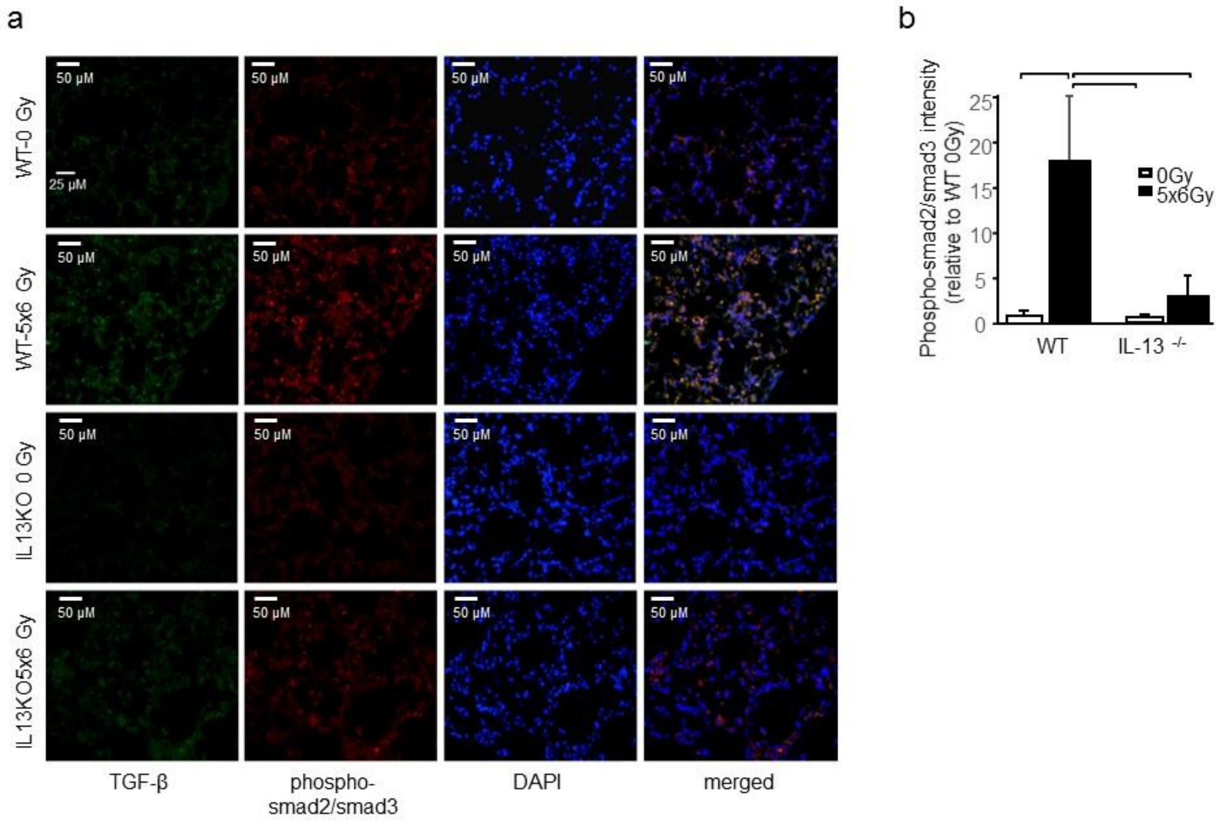

Supplemental Figure 2. Expression of TGF-β and phosphorylated smad2/smad3 in irradiated lungs. Eight to 10 week old female c57BL6/NcR mice were exposed to 5 daily fractions of 6 Gy (5x6Gy) of thoracic irradiation. TGF-β and smad2/smad3 phosphorylation were assessed by immunofluorescence (n=5 mice). **(a)** Representative images are presented. **(b)** The intensity of phosphorylated smad2/smad3 was compared between conditions. Columns: mean, error bars: ± SD, brackets: $p<0.05$; ANOVA with Tukey's correction.

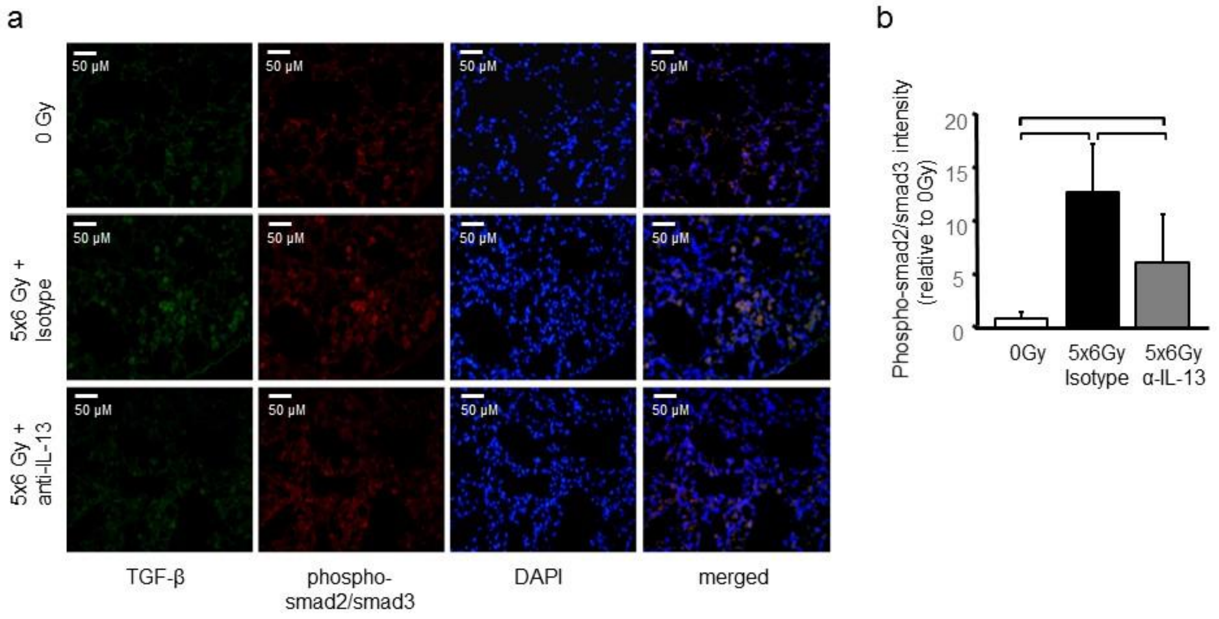

Supplemental Figure 3. Effects of IL-13 neutralization on the expression of TGF-β and phosphorylated smad2/smad3 in irradiated lungs. c57BL6/NcR mice were exposed to 5x6Gy thoracic irradiation and treated with anti-IL-13 IgG or isotype control (0.5mg per mouse) via IP injection. Additional mice were maintained without irradiation (0Gy). Mice were treated weekly for eight weeks starting at 3 weeks post-irradiation, and lung tissue (n=3 mice) was collected 16 weeks following irradiation. TGF-β and smad2/smad3 phosphorylation were assessed by immunofluorescence. **(a)** Representative images are presented. **(b)** The intensity of phosphorylated smad2/smad3 was compared between conditions. Columns: mean, error bars: ± SD, brackets: $p<0.05$; ANOVA with Tukey's correction.

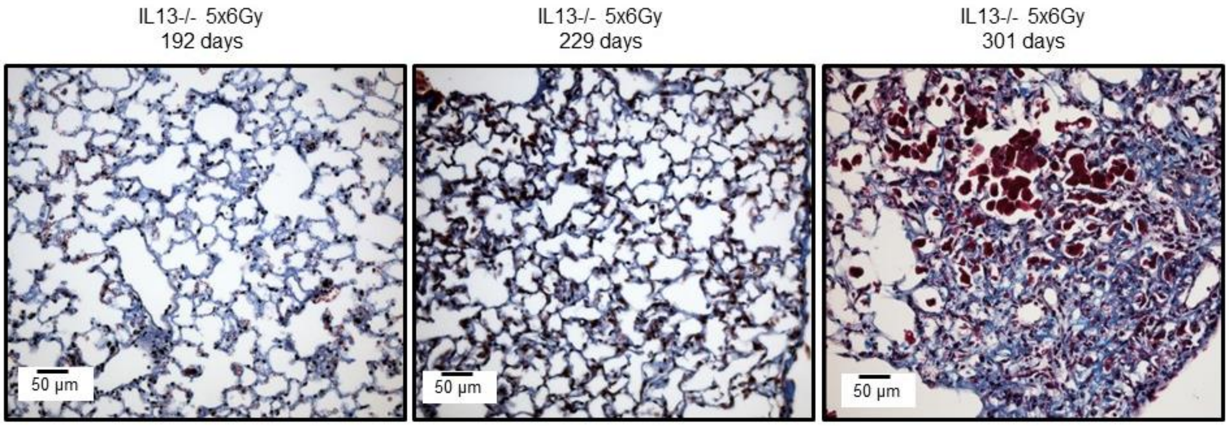

Supplemental Figure 4. Histologic findings in long-term survivors of thoracic irradiation. Eight to 10 week old female IL-13$^{-/-}$ mice were exposed to 5 daily fractions of 6 Gy (5x6Gy) of thoracic irradiation. Mice were followed for survival. At the time of death, lungs were inflated with neutral buffered formalin and processed for histology. Masson Trichrome staining (collagen: light blue, epithelia: red, nuclei: dark blue) of lung tissue of long term survivors demonstrates minimal fibrotic progression at 192 and 229 days after irradiation. Blood pool is noted in the 229 day survivor due to post-mortem collection. Rare, small, pleural based foci of fibrosis were noted in a mouse that survived 301 days after irradiation. Bar: 50 μm.
